# Evaluation of different fermentation processes for use by small cocoa growers in mexico

**DOI:** 10.1002/fsn3.333

**Published:** 2016-01-22

**Authors:** C. Hernández‐ Hernández, Procopio Alejandro López‐Andrade, Miguel A. Ramírez‐Guillermo, Diana Guerra Ramírez, Juan F. Caballero Pérez

**Affiliations:** ^1^ Instituto Nacional de Investigaciones Forestales Agrícolas y Pecuariaskm. 1 Huimanguillo‐CárdenasHuimanguilloAP 17. CP 86400TabascoMéxico; ^2^ Universidad Autónoma Chapingokm 38.5 México‐TexcocoChapingoMéxico

**Keywords:** Beans quality, boxes, Tabasco, temperature

## Abstract

The aim of this research was to evaluate four different cacao (*Theobroma cacao* L.) fermentation conditions and their effect on fermented bean quality, in order to be able to recommend the most suitable condition to producers in the municipality of Huimanguillo, Tabasco, Mexico. Fermentations were carried out in square wooden boxes with capacity for 1000, 300, and 100 kg of fresh beans, as well as a rotary drum with capacity for 500 kg thereof. The fermentation process was carried out for 7 days, and the response variables measured were mass temperature, total soluble solids (TSS), pH, and acidity. The TSS were totally depleted after 2 days, during which time the yeasts transformed them into ethanol at temperatures of 25–35°C. The most notable temperature increase in the four treatments was 49°C on the third day, corresponding to a decrease in pH from 6.31 ± 0.40 to 4.76 ± 0.03 and an increase in acidity from 0.38 ± 0.04 to 1.17 ± 0.25 g kg^−1^, due to the formation of organic acids. There were no significant differences among the four treatments (Tukey *α *= 0.05). The cut test showed that fermentation in 300‐ and 100‐kg boxes and in the 500‐kg rotary drum produced the same effect on fermentation quality, but the 1000‐kg boxes exhibited lower quality (Tukey *α *= 0.05).

## Introduction

In cocoa, the precursor compounds that give rise to the flavor and aroma attributes develop during the bean fermentation, drying, and roasting stages Rohan and Stewart 1966;. Fermentation is a very important step in which the successive action of yeasts, acetic acid bacteria, and lactic acid bacteria are involved Ardhana and Fleet 2003; Camu et al. 2007; Biehl et al. 1985; Sandhya et al. 2016;.These microorganisms act on sugars and acids in the cocoa pulp, triggering intense enzymatic activity to hydrolyze sugars, organic acids, proteins, and polyphenols present in the beans Ardhana and Fleet 2003; Lehrian and Patterson 1983;. This microbial load is directly related to the conditions prevailing in the fermentation mass such as pH, temperature, and the presence of oxygen Camu et al. 2007; Sandhya et al. 2016.

The yeasts, of which *S. cerevisiae* is the most important, dominate the mass during the first 24 h. They are responsible for converting the pulp sugars into ethanol under anaerobic conditions Camu et al. 2007; Puziah et al. 1998;. In a Mexican research about yeasts predomination, the results showed that *Pichia kudriavzevii*, S*. cerevisiae, Saccharomycopsis crataegensis,* and *Hanseniaspora guilliermondii* were the predominant yeasts Arana‐Sánchez et al. 2015;. The subsequent total depletion of the mucilage substrates, which are sugars and acids, leads to the next phase of the process which is lactic acid fermentation, which is favored by manual removal of the fermentation mass done to facilitate greater oxygen penetration, thereby causing the aerobic condition which leads to the presence of lactic acid and acetic acid bacteria, which oxidize ethanol to lactic and acetic acid, respectively Schwan and Wheals 2004; however, the later studies had demonstrated that there are acetic acid production without ethanol production during fermentation process, suggesting acid acetic production from lactic acid bacteria Ho et al. 2014;. In addition, citric, oxalic, phosphoric, succinic, and malic acids are also produced Camu et al. 2007; Puziah et al. 1998;. Due to the exothermic nature of the above reactions, the temperature within the boxes rises up to 50°C; under this condition, cotyledon cracking, and embryo death occur due to the penetration of ethanol and organic acids, resulting in a decrease in internal pH and internal damage to the structure of the cocoa bean Lehrian and Patterson 1983; Schwan and Wheals 2004; Hii et al. 2006;. During this phase, flavor and aroma precursors develop and pigments are degraded by the activity of endogenous enzymes such as invertases, glycosidases, proteases, and polyphenol oxidases Biehl et al. 1985; Brito et al. 2000; Hansen et al. 1998; Misnawi et al. 2003;. Other microbial metabolites such as esters and pyrazines may enter the bean cotyledon and act as flavor precursors or directly as flavor compounds Puziah et al. 1998;. As a result of these biochemical reactions, numerous compounds such as reducing sugars, peptides, and amino acids, which are subsequently modified by Strecker degradation and Maillard reactions during the drying and roasting of the beans, are also produced Hansen et al. 1998; Misnawi et al. 2003, 2004; Crafack et al. 2014.

Fermentation is influenced by several factors, among which the most important are the state of maturity in which the pod is harvested, time that it is left stored prior to fermentation, De Bertorelli et al. 2009 type of cocoa, Lemus et al. 2002 fermentation method, Contreras et al. 2004; de Fariñas et al. 2003; Portillo et al. 2005; Vargas et al. 1989 and bean removal frequency, as a higher frequency enables better fermentation.Puziah et al. 1998; Portillo et al. 2005; Schwan et al. 1990; Senanayake et al. 1997 In a study conducted to compare fermentation masses ranging from 10 to 100 kg, fermentation with 60‐kg masses was recommended because it provides the highest concentration of flavor precursors, such as total reducing sugars Puziah et al. 1998.

In the cocoa‐growing region of the state of Tabasco, Mexico, cocoa fermenting, and drying is carried out in collection plants using wooden containers with capacity to ferment approximately one ton of fresh beans. In order to propose fermentation methods that could be used at the level of individual producers, we assessed the efficiency of fermentation in 1000‐, 300‐, and 100‐kg boxes, as well as a 500‐kg rotary drum. The relationship between each treatment and bean quality was also determined.

## Materials and Methods

### Plant material

A mixture of cocoa (*Theobroma cacao* L.) beans from the 2010–2011 crop production cycle was used. The beans were acquired from the local cocoa producers' association in Huimanguillo, located in the state of Tabasco, Mexico.

### Cocoa bean fermentation

Fermentation was carried out at the fermentation facilities of the local cocoa producers' association in Huimanguillo, located in the state of Tabasco, Mexico. Pods were harvested and immediately opened, then beans were extracted and fermentation was started. The fermentation process was conducted in square boxes and a rotary drum built with melina wood. Each treatment was carried out simultaneously with three replications. The dimensions of the boxes for 100 kg of beans were 50 × 50 × 50 cm, the dimensions of the boxes for 300 kg were 75 × 75 × 75 cm and those of the boxes for 1000 kg were 100 × 100 × 120 cm. The wooden rotary drum was a cylinder designed with a capacity for 500 kg; its dimensions were 97 cm in diameter and 150 cm in length. The wooden boards that form the cylinder have 20 cm width and 2.5 cm thick. The containers were filled with fresh beans and once draining stopped, they were removed every 24 h from that time on. Boxes were kept covered with a jute lid to prevent heat escape. Fermentation lasted a total of 7 days.

### Sampling

1 kg samples of each treatment were taken every 24 h and dried to an approximate moisture content of 7%. Drying was carried out in the sun with removals every 2 h to promote aeration and prevent molding on the beans. The variables evaluated were fermentation mass temperature, pH, and titratable acidity of the cotyledon of the dried beans using the modified Helrich (1990) method for cocoa beans and cut test (% of purple, slaty, and brown beans).

### Temperature monitoring

The temperature of the fermentation mass and the surrounding temperature were recorded during the 7 days of fermentation using a Hanna Instruments temperature monitor.

### Ph and titratable acidity

pH and titratable acidity were determined in cotyledon according to the methods established by the Helrich (1990). The pH reading was taken with a potentiometer at 22°C. For the determination of titratable acidity, a 50–mL solution of homogenized sample was taken and titrated at pH 8.3 with a NaOH 0.1 N solution. Data are reported in % acetic acid.

### Cut test

To determine the degree of fermentation, the cut test was performed with a Magra cutting unit (Model 12, TESERBA Technischer Service, Herrliberg, Switzerland), with which the violet, slaty, and brown beans were classified. The percentage was calculated using the cut test score (CTS) as shown belowPuziah et al. 1998; Hii et al. 2006, 2011:

$$\begin{array}{cc} \text{Cut test score}\;=\; & \left(10\times \%\;\mathrm{brown}\right)\\ & +(5\times \%\;\mathrm{partly}\;\mathrm{purple}/\mathrm{brown})\\ & +(0\times \%\;\mathrm{purple}\;\mathrm{and}\;\mathrm{slaty}) \end{array}$$

### Data analysis

A completely randomized experimental design with three replications was used. Statistical analyzes were performed by analysis of variance and comparison of means with Tukey's test (*α *= 0.05) using SAS version 9.2 software for Windows (Cary, NC, USA).

## Results and Discussion

**Figure 1:**
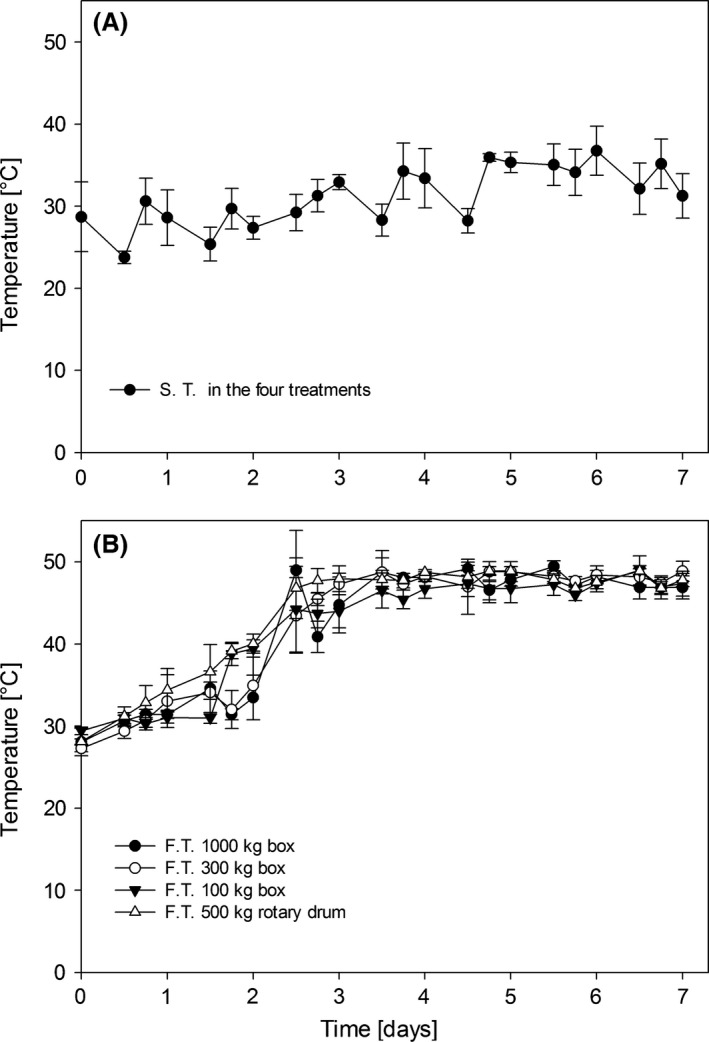
(A) Surrounding temperature (S.T.) in the four treatments. (B) Changes in temperature during the fermentation of the four treatments without significant differences (*α *= 0.05). F.T. (Fermentation temperature) S. T. (Surrounding temperature in the four treatments).

Figure 1A shows the surrounding temperature of the four treatments, which ranged between 25 and 35°C, whereas Figure 1B shows the progress of the temperature at five points inside the containers throughout the fermentation process. During the first 2 days the mass temperature ranged between 25 and 40°C; by the third day the temperature rose to 48 ± 1.054°C, remaining constant until the end of the process without significant differences among treatments (*α *= 0.05). This behavior indicates that in the fermentation process the temperature of the mass is not affected by increasing the amount of cocoa or by the rotation. The temperature rise is caused by the energy released in the exothermic reaction of the conversion of ethanol into acetic acid by acetic acid bacteria Nielsen et al. 2007; this causes, in addition to the death of the embryo, changes in the tissue structure of the cotyledon Camu et al. 2007, 2008; Samah et al. 1993; Senanayake et al. 1995;. In some studies, simultaneous development of yeasts, lactic acid bacteria, and acetic acid bacteria has been found Camu et al. 2007.

**Figure 2:**
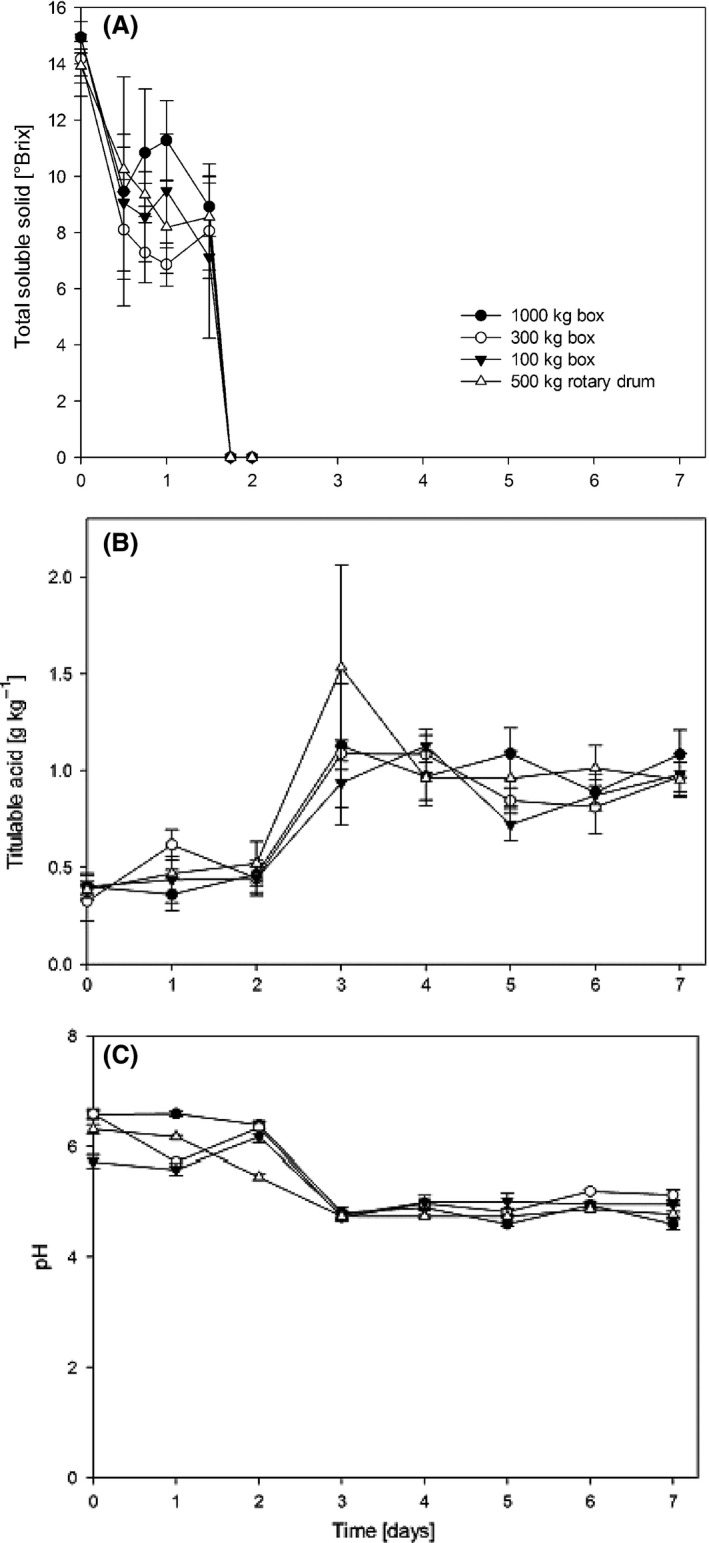
Behavior of total soluble solids, pH and titratable acidity during fermentation in the four treatments.

Figure 2 shows the recorded changes in pH, TSS, and the acidity of the cotyledon in the dried beans during the fermentation process. The TSS are a way to quantify the total sugars of the samples. During the first 2 days of fermentation, their total depletion due to the action of the yeasts that convert these pulp solids into ethanol is observed (Fig. 2A). In this first phase of fermentation, acidity and pH are constant and temperatures are below 40°C. The second phase of fermentation involves the transformation of the ethanol into organic acids (acetic and lactic) due to the activity of lactic acid and acetic acid bacteria; Camu et al. 2007; Biehl et al. 1985; Misnawi et al. 2003; Camu et al. 2008 as a result of this, on the third day of fermentation the titratable acidity increases from 0.38 ± 0.04 to 1.17 ± 0.25 g kg^−1^ (Fig. 2B) and the pH decreases from 6.31 ± 0.40 to 4.76 ± 0.03 (Fig. 2C). This exothermic reaction is what causes the increase in temperature on the third day Nielsen et al. 2007. The TSS, pH, and titratable acidity showed no significant differences among the treatments (Tukey *α *= 0.05).

Acidification due to the presence of acetic acid during fermentation leads to several biochemical changes such as generation of peptides and amino acids from reserve proteins by action of proteases of the seed and reducing sugars, precursor compounds of the Maillard reactions which occur during drying and roasting of the beans Rohan and Stewart 1966;. According to Jinap et al. (2008), during the first 3 days of fermentation there is high proteolytic activity which increases the amount of free amino acids. Biehl and Passern (1982) have reported that the optimum protease activity of cocoa is from ph 4.5 to 5.5, a condition favored from day two of fermentation in this study (Fig. 2).

**Figure 3:**
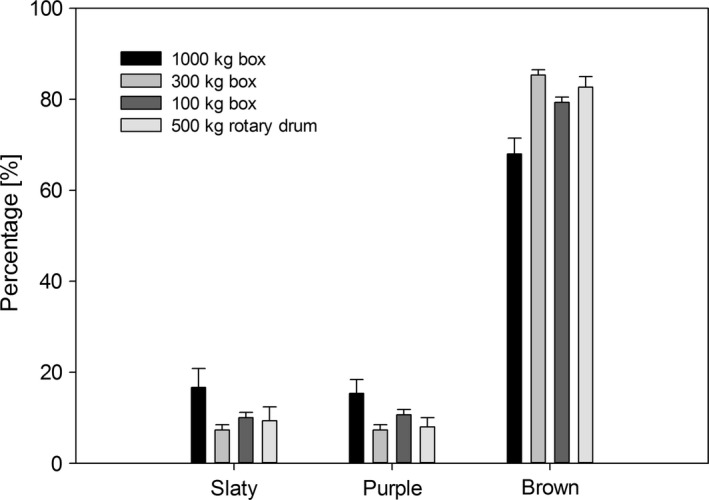
Final fermentation quality in beans of the different treatments.

One of the fermentation degree used to determine the final price of cocoa is the cut test. This is a measure of the oxidation of anthocyanins in cocoa beans in which gray and purple beans are characteristic of a poorly fermented bean and brown beans are typical of good fermentation. At the time fermentation was stopped (day seven), 68% brown beans were obtained in 1000‐kg boxes, 85.33% in 300‐kg boxes, 79.33% in 100‐kg boxes, and 82.66% in the rotary drum (Fig. 3). These changes in the degree of fermentation during the process are the result of reductions in the concentration of polyphenols due to their spread outward from the beans, and their subsequent oxidation and condensation. Polyphenol oxidase is responsible for catalyzing o‐difenol to o‐quinon oxidation reactions; o‐quinones are responsible for the brown color of the beans. The optimum temperature for polyphenol oxidase activity is from 42 to 45°C Misnawi et al. 2002, temperatures which correspond to those reached on day three of the fermentation process.

**Figure 4:**
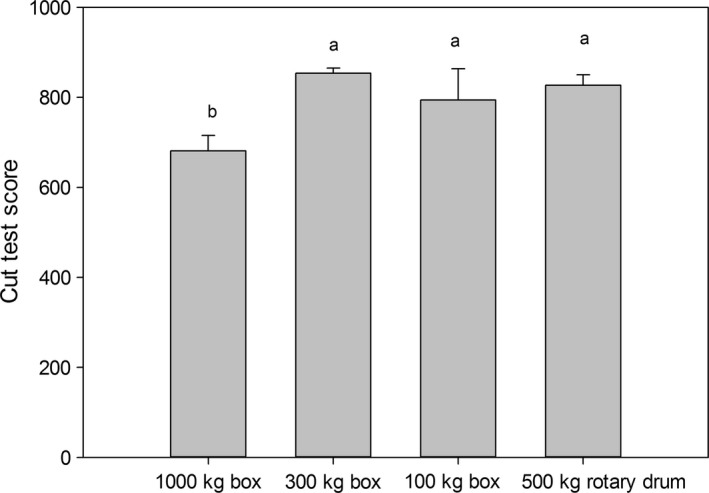
Cut test score in the different treatments.

The cut test score (Fig. 4) showed that by day seven the treatments involving 300‐kg boxes, 100‐kg boxes, and the 500‐kg rotary drum produced the same effect on the degree of fermentation, as no significant differences (Tukey *α *= 0.05) were found among them; however, in 1000‐kg boxes the cut test score is lower than in the other treatments. Lower values obtained in 1000 kg boxes could be that in some parts of the box farthest from the center the temperature and pH conditions were not suitable for optimal fermentation. The cut test values were 681.13, 853.76, 794.03, and 827.15 in 1000‐, 300‐, and 100‐kg boxes, and in the 500‐kg rotary drum, respectively. These cut test values are acceptable; Hii et al. Hii et al. 2011 obtained values from 333.3 to 950 in different cocoa samples.

## Conclusions

According to the parameters evaluated in this research, the fermentation conditions are conducive to obtaining good physicochemical fermented bean quality in both 1000‐kg, 300‐kg, and 100‐kg square wooden boxes and a 500‐kg rotary drum. The cut test score showed that fermentation in 300‐kg boxes provides the best final fermented bean.

## Conflict of Interest

None declared.
